# A Wide Dynamics and Fast Scan Interrogating Method for a Fiber Bragg Grating Sensor Network Implemented Using Code Division Multiple Access

**DOI:** 10.3390/s120505888

**Published:** 2012-05-08

**Authors:** Youngbok Kim, Sie-Wook Jeon, Won-Bae Kwon, Chang-Soo Park

**Affiliations:** Gwangju Institute of Science and Technology (GIST), 1 Oryong-dong, Buk-gu, Gwangju 500-712, Korea; E-Mails: youngbokk@gmail.com (Y.K.); pinemint@gist.ac.kr (S.-W.J.); wbkwon@gist.ac.kr (W.-B.K.)

**Keywords:** optical sensor network, code division multiple access, fiber Bragg grating sensor, wavelength-to-time conversion, sliding correlation

## Abstract

We propose and demonstrate a fiber Bragg grating (FBG) sensor network employing the code division multiple access (CDMA) technique to identify information from individual sensors. To detect information without considering time delays between sensors, a sliding correlation method is applied, in which two different signals with the same pseudo-random binary sequence (PRBS) pattern, but slightly different frequencies, are applied to the source and detector sides. Moreover, for time domain detection, a wavelength-to-time conversion technique using a wavelength dispersive medium is introduced. The experimental results show that the proposed sensor network has a wide strain dynamic range of 2,400 με and a low crosstalk of 950:1.

## 1. Introduction

As one of the most promising strain and temperature sensors, the fiber Bragg grating (FBG) has attracted considerable attention due to its novel properties such as small size, fiber compatibility, immunity to electromagnetic interference, and multipoint sensing capability [1,2]. To implement a multiple FBG sensor network, various methods such as wavelength division multiple access (WDMA), time division multiple access (TDMA), and code division multiple access (CDMA) have been reported. The primary advantage of using an FBG as a sensor is that a large number of sensors can be integrated along a single fiber [3]. In the WDMA system, the maximum number of sensors is determined by the usable spectral bandwidth of the system and the wavelength shift of each sensor [4]. The TDMA system detects by reusing the spectrum of the pulse source, and the pulse has to travel to the last FBG sensor in the sensor array and reflect back before another pulse can be sent from the source. This imposes a limit on the sensor detection speed, number of sensors, and maximum distance between sensors [5]. The CDMA system utilizes the autocorrelation property to separate individual sensors from the multiplexed output of many sensors [6]. To identify each sensor, a pseudo-random binary sequence (PRBS) code is sent as a random signal into the FBG sensor array, and the information of the corresponding sensor is decoded in the electrical domain [7].

In this paper, we report a CDMA sensor network based on a sliding correlation method [8,9] that provides a higher scanning rate than a conventional CDMA scheme [7]. Further, a wavelength-to-time conversion technique is introduced to provide a wide dynamic range to FBG sensors. This conversion is implemented using a wavelength dispersive medium, so the change in the center wavelength of each sensor is converted into a time shift in the correlation peak. Moreover, by using a wideband reflective semiconductor optical amplifier (RSOA) as an optical source, WDMA can be integrated with FBG sensors having different center wavelengths.

## 2. Proposed CDMA Sensor Network

The basic concept of a proposed FBG sensor network system is to use a wavelength shift caused by external strain. The grating period of the FBG is changed when the external strain is applied to the sensor. As a result, the center wavelength of the reflected light is also changed. Depending on the distance between FBG sensor and photodetector (PD), the incoming sensor information has a time delay and it is merged into the PD through the wavelength-to-time converter, which changes the wavelength shift into a time shift. For a CDMA application based on a correlation technique, an electrical signal with a random data pattern is split into two signals: one is applied to the optical source and the other is applied to the mixer at the detector to obtain the autocorrelation peak. In conventional CDMA, time delays from all sensors should be compensated for before mixing. This makes it difficult to expand a system to accommodate new sensors in a practical application. To overcome this, a sliding correlation method is employed in the proposed sensor system.

**Figure 1.**
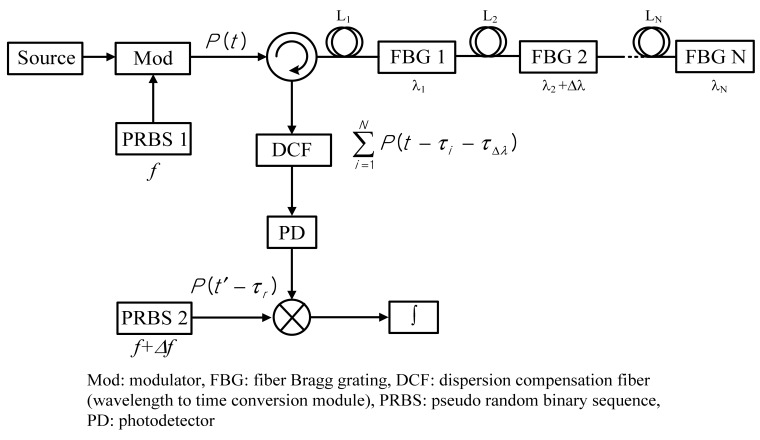
Conceptual diagram of FBG-based sensor network using sliding correlation and DCF as a function of wavelength-to-time conversion.

A proposed conceptual diagram of a CDMA sensor network using sliding correlation is shown in Figure 1. To cover the whole wavelength band of all sensors, a broadband light source is used. Then, we apply two different random signals, with the same data patterns, but slightly different frequencies, to the optical modulator and the mixer. This slight difference in frequency has a scanning effect and thereby gives an autocorrelation output without any information about the time delay between the two signals fed towards the PD. As a result, whenever sensors are added to the network, the time delays from the source to the mixer need not be measured. Moreover, the scanning speed depends on the frequency of the random signal, and high-speed scanning to all sensors is thus possible. The optically modulated signal is applied to each sensor located at different places (here, L_1_ to L_N_). Then, the wavelength band corresponding to each sensor is selected by each FBG and enters the dispersion compensation fiber (DCF) as a wavelength-to-time conversion module of dispersive medium. The dispersive medium shows different propagation velocities according to the wavelength of the light. When a strain is applied to FBG 2 as shown in Figure 1, the corresponding wavelength shift (Δλ) is converted into a time shift (τ_Δλ_) in the appearance time of the autocorrelation peak. Finally, by reading the difference in arrival time, this technique can provide a wider dynamic range than the conventional methods reading the peak value.

In general, a narrow optical source like a distributed feedback laser diode (DFB-LD) can be used in a CDMA sensor network. However, in this case, the maximum number of FBG sensors installed in a serial line is limited by the transmission coefficient of each FBG. We can slice the spectrum of the DFB-LD and assign one portion to each sensor. Nevertheless, this has a limitation in the available sliced spectrum. Furthermore, narrow wavelength spacing between the sensors might induce channel crosstalk. Therefore, we use a broadband light source like an RSOA to secure a large dynamic range according to the wavelength change. RSOA is a special type of semiconductor optical amplifier (SOA), which has a high reflective (HR) coating on one facet and an anti-reflective (AR) coating on the other facet to produce a highly versatile gain medium. Although its waveguide structure is similar to a conventional SOA, the RSOA has a lot of different optical properties, such as low noise figure and high optical gain at low drive current. The scanning time of proposed sensor network is determined by:

(1) $$T_{S}=\frac{n}{\Delta f}$$

where n is the bit length of the PRBS code and Δf is the frequency difference between the two PRBSs applied to the source and the mixer. Additionally, the autocorrelation pulse width is defined as:

(2) $$T_{W}=\frac{2}{\Delta f}$$

Therefore, the maximum number of sensors available without inter-symbol interference is limited by the optical source bandwidth and the PRBS pattern length 2Ts/Tw (=n).

The scanning effect associated with the sliding correlation can be explained as follows: to prevent autocorrelation of the unwanted pattern, a random signal (here a PRBS) is used. In a PRBS signal, the probability of repeating the same pattern within one period of scan is almost zero. Hence, insofar as the reflected lights from all sensors are within one period of scan, each sensor can be identified with a random pattern. Moreover, by giving a slightly different frequency to one (here connected to the mixer), a scanning effect is given to each sensor.

## 3. Experiment and Results

### 3.1. Experimental Setup

**Figure 2.**
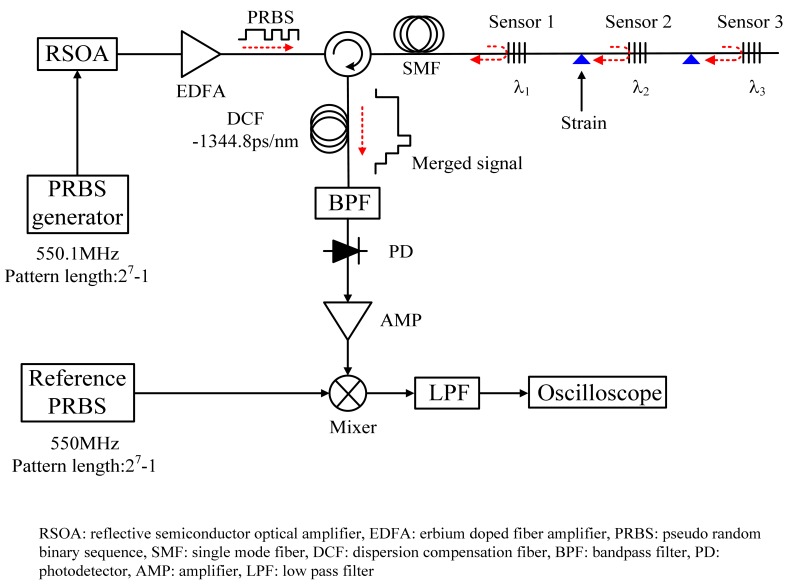
Experimental setup.

Figure 2 shows the experimental setup to investigate the performance of the proposed FBG sensor network. For sliding correlation, two PRBS signals with the same data pattern but different frequencies of 550.1 Mbps and 550 Mbps are applied to the RSOA and the mixer, respectively. These signals can be generated from two pattern generators (Anritsu MP1763C), which are operated by different external clock sources but the same code program.

Also, is not necessary to have autocorrelation in the the phase control between two signals. The bandwidth and maximum output power of the RSOA are >30 nm and −15 dBm at 1,550 nm, respectively. The RSOA as a broadband source is driven at a bias current of 55 mA and directly modulated by the PRBS data pattern with a pattern length of 127 (= 2^7^ − 1) from the pulse pattern generator (PPG). Another PRBS pattern is applied to the mixer. The modulated signal of the RSOA is amplified using an erbium-doped fiber amplifier (EDFA) and transmitted into the FBG sensor array through an optical circulator. From the conceptual diagram of Figure 1, the parallel FBG sensor is applied to each sensor, but the proposed serial FBG sensor also could be applied to each sensor because it is located at different place from optical source. In this experiment, three FBGs with different center wavelengths but the same reflectivity was used for a simple demonstration of applied strain. The center wavelengths of the sensors were 1,550.9, 1,552.6, and 1,555.8 nm, respectively. The bandwidth of each sensor was 0.32, 0.3, and 0.26 nm and the reflectivity of each sensor was 99%.

For an input signal with a broadband spectrum, only the wavelength matched to the Bragg wavelength of the FBG is reflected back at the FBG. Wide wavelength spacing between sensors is helpful to achieve wide dynamic range, because small wavelength spacing results in wavelength crosstalk. Due to the time delay corresponding to the location of each sensor, the signals arrived at the output of the circulator have different data patterns. To convert the wavelength shift into a time shift, the combined signals enter a DCF with a dispersion coefficient of −1,344.8 ps/nm at 1,550 nm, and the conversion ratio is determined by the dispersion coefficient of the DCF. When we consider the resolution of the electrical devices used to read the variation, the conversion ratio influences the sensitivity of the sensor network. After the DCF, the optical signal is converted into an electrical signal by the PD. Then, the electrical signal is correlated with the reference PRBS pattern.

### 3.2. Results and Discussion

**Figure 3.**
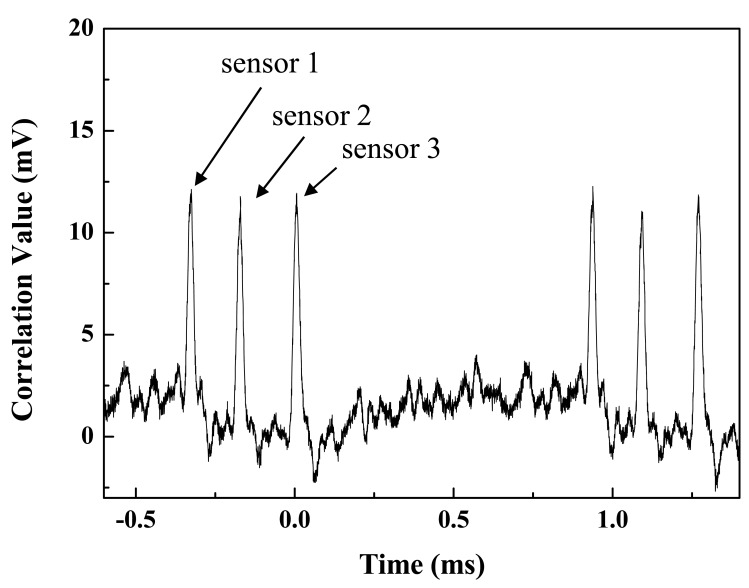
Autocorrelation trace of each sensor.

The autocorrelation traces after the mixer and low-pass filter are displayed in Figure 3. With no strain, each autocorrelation trace has a peak at the given time; i.e., with the given time delay. The latter three peaks repeat the first three peaks. If we use a PRBS with a longer pattern length, more sensors can be accommodated. The variation in peak value is due to differences in reflectivity and the wavelength-dependent optical loss of each sensor. The scanning time and pulse width were measured as 1.26 ms and 0.02 ms, respectively, which matched the values from Equations (1) and (2) Thus, the maximum number of sensors is determined by n (here n = 127) without inter-symbol interference between autocorrelation pulses. Compared to conventional CDMA technology based on the correlation method, the sliding correlation method makes it possible to scan all sensors in a rapid time [7]. The proposed CDMA method was compared with other multiplexing methods in terms of scanning time, potential number of sensors, and demodulation method [10] and it is summarized in Table 1.

**Figure 4.**
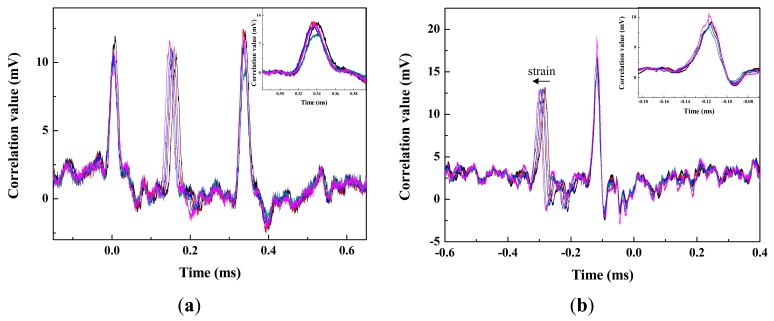
(**a**) Measured autocorrelation pulses. (**b**) Improved autocorrelation traces after using the BPF.

Next, to investigate deviation in the time domain, we applied external strain to sensor 2 and measured its autocorrelation peak. The wavelength of sensor 2 was shifted from 1552.5 nm to 1555.1 nm. As shown in Figure 4(a), the wavelength shift of the sensor appeared to be a time shift of the autocorrelation peak, showing a left side shift along the time axis. The sensitivity of the time shift depends on the dispersion coefficient of the DCF. In Figure 4(a), the small time variation in the autocorrelation trace of sensors 1 and 3 are caused by the wavelength crosstalk between neighboring sensors. The optical source has a broadband spectrum and the output is amplified by using an EDFA with amplified spontaneous emission noise over a wide wavelength range. Thus, the wavelength band selected by FBG 2 has a tail in spectrum. If the center wavelength is changed by the applied strain, then the tail profile is also shifted and induces crosstalk to the neighboring sensor's spectrum. These time variation can be removed by using an optical band-pass filter (BPF) to suppress the inter channel crosstalk. Figure 4(b) shows the reduced time variation of autocorrelation trace after using the BPF. Each inset of Figure 4 shows time variation of sensor 3 without and with BPF. The time variation of autocorrelation peak is about 6 μs from the inset of Figure 4(a), but after using the BPF the variation was reduced into 0.02 μs as shown in the inset of Figure 4(b).

**Figure 5.**
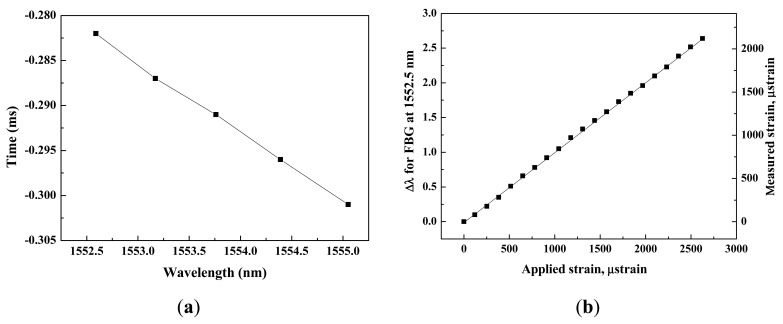
(**a**) Time shift curve for sensor 2; (**b**) Measured strain and wavelength shifts against applied strains for sensor 2.

Figure 5(a) shows the relation between the time shift and the strain produced in sensor 2. The instant of the time of autocorrelation peak shifted linearly as the strain in sensor 2 increased, because of the negative dispersion coefficient of the DCF. When the wavelength of sensor 2 shifted from 1552.5 nm to 1555.1 nm, its time of autocorrelation peak shifted from −0.282 ms to −0.301 ms. The conversion ratio between shifted time and wavelength was apparently −0.007 ms/nm. Furthermore, a time variation of sensor 3 is 0.02 μs when the shifted time of sensor 2 is 19 μs from applied strain. Therefore, the error rate by crosstalk between neighboring sensors is 950:1. Figure 5(b) shows the spectral shifts and the measured strain of the sensor 2 against applied strains for sensor 2. The strain dynamic range and transfer efficiency were obtained 2400 με and 0.81, respectively.

Table 2 shows a summary about the relationship between dynamic range and time variation with the increasing bandwidth of BPF. We can reduce time variation further by taking narrower bandwidth. However, the limitation in wavelength shift due to narrow bandwidth, *i.e.*, power loss cut out by the edge side of the filter shape, decreases the achievable dynamic range.

## 4. Conclusions

A FBG sensor network based on CDMA with a brief scanning time and wide spectral dynamic range was proposed and demonstrated. A sliding correlation method facilitates continuous and rapid monitoring without considering time delays among the FBG sensors. The scanning function was implemented using two PRBS signals with the same data pattern, but different frequencies. The scanning time is determined by the pattern length. The dynamic range is determined by the bandwidth of the optical source used. For signal detection in the time domain, a dispersive medium like a DCF is used. This DCF has a negative dispersion coefficient, and so the proposed scheme has a lead or lag characteristic in the time domain with the increase or decrease in the applied strain, revealing the directivity in the variation of the strain produced. The proposed sensor network provides wide dynamic range of over 2400 με and low crosstalk of 950:1.

## Figures and Tables

**Table 1.** Summary of properties between proposed CDMA method and other multiplexing methods.

**Multiplexing method**	**Proposed CDMA**	**Conventional CDMA**	**WDMA**	**TDMA**
**Scanning time (ms)**	1.26	13	1 (few KHz)	0.01 (few hundred KHz)
**Potential number of sensors**	100	100	16	100
**Demodulation method**	Sliding correlation	Correlation	Interferometric/Scanning Febry-Perot	Edge-filter/Interferometric

**Table 2.** Summary of the relationship between dynamic range and time variation with the increasing bandwidth of BPF.

**Bandwidth of the BPF (nm)**	**Dynamic Range (με)**	**Time variation (μs)**
2.5	2400	4.9
2.0	1924	2.4
1.5	1435	1.1
1.0	967	0.25
0.5	475	0.03
0.1	95	0.002
